# Impact of *TLR4* and *MYD88* Genetic Variants on Disease Progression and Prognosis in Laryngeal Squamous Cell Carcinoma

**DOI:** 10.3390/ijms27114760

**Published:** 2026-05-25

**Authors:** Akvilė Mikulskienė, Roberta Vadeikienė, Aurelija Vegienė, Rasa Ugenskienė, Elona Juozaityte, Evaldas Padervinskis

**Affiliations:** 1 Department of Otorhinolaryngology, Lithuanian University of Health Sciences, 50161 Kaunas, Lithuania; akvile.mikulskiene@lsmu.lt (A.M.); aurelija.vegiene@lsmu.lt (A.V.); evaldas.padervinskis@lsmu.lt (E.P.); 2 Oncology Research Laboratory, Institute of Oncology, Lithuanian University of Health Sciences, 50161 Kaunas, Lithuania; rasa.ugenskiene@lsmu.lt; 3 Department of Genetics and Molecular Medicine, Lithuanian University of Health Sciences, 50161 Kaunas, Lithuania; 4 Institute of Oncology, Lithuanian University of Health Sciences, 50161 Kaunas, Lithuania; elona.juozaityte@lsmu.lt

**Keywords:** SNP, *TLR4*, *MYD88*, laryngeal cancer

## Abstract

Laryngeal cancer is a relatively uncommon malignancy with predisposing genetic factors that remain unclear. Single-nucleotide polymorphisms (SNPs) in genes involved in innate immune signaling may contribute to the development and progression of laryngeal carcinoma. This study aimed to evaluate the association of *TLR4* (rs7037225, rs11536889, rs7037117) and *MYD88* (rs7744, rs6853) polymorphisms with the risk of laryngeal squamous cell carcinoma (LSCC), as well as its clinical and pathological characteristics and survival. A retrospective case–control study involving 172 LSCC patients and 220 healthy controls was conducted. Genotyping was performed using real-time PCR from venous blood samples. *MYD88* rs7744 was significantly associated with tumor size and lymph node involvement. Survival analysis showed a significant association between *rs7744* and recurrence-free survival (RFS), with the AG and GG genotypes linked to poorer outcomes. Conversely, carriers of the *TLR4* rs7037225 CT genotype showed significantly improved RFS, with *p* ranging from 0.024 to 0.037 across models. Considering the significant roles of *TLR4* and *MYD88* in Toll-like receptor signaling, these findings may reflect the involvement of innate immune pathways in LSCC progression. In summary, *MYD88* rs7744 was associated with clinicopathological features and RFS, while *TLR4* rs7037225 appeared to have a potential protective effect on survival.

## 1. Introduction

Laryngeal cancer involves tumors of the supraglottic, glottic, and subglottic subsites. In 90–95% of cases, laryngeal cancer arises from the mucosal epithelium as squamous cell carcinoma (LSCC). In contrast, non-squamous cell cancers, such as adenocarcinomas, neuroendocrine tumors, sarcomas, lymphomas, and minor salivary gland tumors, are rarely diagnosed. According to the latest WHO/IARC GLOBOCAN estimates for 2021, there were approximately 200,900 new cases of laryngeal cancer and 117,250 deaths globally, ranked among the top 20 cancers worldwide in both incidence and mortality [1].

In recent years, significant advances have been made in the treatment of LSCC patients; new biological and immunomodulatory therapies are improving relapse-free and overall survival. Unfortunately, early diagnosis remains problematic due to the lack of specific biomarkers that could help primary care physicians detect disease early and enable the most effective treatment. However, this is not the case; advanced-stage LSCC is diagnosed in the majority of patients, and overall survival to this day is around 50–70%. This number is drastically lower when distant metastases are present at the time of the diagnosis, decreasing to around 30–48%, with the poorest being the supraglottic type (according to the American Cancer Society SEER data) [2,3].

Many epidemiological studies have demonstrated that tobacco consumption is strongly associated with increased risk of LSCC, with a 10–15-fold higher risk in smokers compared with non-smokers, and an even greater risk among heavy smokers, reaching up to a 40-fold increase [4,5]. Despite the widespread prevalence of smoking (according to WHO data from 2020, approximately 1.25 billion adults use tobacco), laryngeal cancer remains relatively rare, with an incidence rate of around 2.0–2.3 new cases per 100,000 individuals per year [6,7]. These observations indicate that additional genetic and epigenetic factors may contribute to disease development alongside known risk factors.

Small genetic changes, even at the nucleotide level, can impair protein function and, therefore, reduce protection against environmental factors. Single-nucleotide polymorphisms (SNPs) are the most common genetic variants and are present in >1% of the population. Most are benign, although some variants are associated with increased risk of various diseases, including cancer [8]. One widely accepted concept is that impaired immune responses lead to chronic inflammatory processes in the body, which are tightly linked to cancer development. Therefore, pathways involved in innate immunity activation and mutations in their regulatory genes are being intensively researched to better understand individual susceptibility to cancer.

The Toll-like receptor (TLR) family is considered a key group of pattern recognition receptors because they detect pathogen-associated molecular patterns (PAMPs), components of various microorganisms, including bacteria, viruses, and parasites. However, TLRs can also be activated by endogenous molecules, such as HMGB1, S100 proteins, and histones, released during tissue damage and cell death and referred to as damage-associated molecular patterns (DAMPs) [9]. Recognition of these molecular patterns is a vital step in activating innate immunity and contributes to protection against infections, promotion of chronic inflammation, and subsequent cancer development.

To date, 10 Toll-like receptor types have been identified in humans. There are two main groups of TLRs based on their localization: those located on the plasma membrane (TLR1, TLR2, TLR4, TLR5, TLR6, and TLR10) or those localized to endosomes and lysosomes (TLR3, TLR7, TLR8, and TLR9). Cell surface TLRs primarily recognize extracellular lipids and proteins, while endosomal TLRs detect microbial and endogenous nucleic acids released during host cellular damage. TLR consists of the ectodomain (important for PAMP recognition), the transmembrane part, and a cytoplasmic Toll/IL-1 receptor (TIR) domain that launches further downstream signaling. When the ectodomain recognizes PAMPs or DAMPs, the TIR component uses adaptor proteins, primarily Myeloid Differentiation Primary Adapter–Response 88 (MyD88) and TIR domain-containing adapter-inducing interferon-β (TRIF), which mediate downstream signaling and drive the production of cytokines, type I interferons, and chemokines, thereby activating the innate immune system. One of the most researched Toll-like receptors is TLR4, the main receptor for lipopolysaccharides (LPSs), present in the outer membrane of Gram-negative bacteria. TLR4 expression is detected in immune cells (such as monocytes, macrophages, and dendritic cells). Reduced levels are found in epithelial and endothelial cells, as well as in various other cell types. Conversely, TLR4 is highly expressed in numerous cancer cell types and is associated with inflammation. Unlike most TLRs, TLR4 can utilize both MyD88 and TRIF pathways as opposed to other TLRs using only MyD88 downstream signaling [9,10].

Impaired balance in the activation, maintenance, and regulation of the immune response is thought to underlie the association of TLR4 and MyD88 with inflammatory diseases and tumors. TLR4 is associated with the development of various autoimmune diseases, including systemic lupus erythematosus, systemic sclerosis, psoriasis, and rheumatoid arthritis [11,12,13,14]. Additionally, research on *TLR4* gene polymorphisms has found associations with increased incidence of several cancers, including prostate [15], lung [16], breast [17], gastric [18], and colorectal [19]. On the other hand, a protective role has been observed in the development of hepatocellular cancer [20]. Furthermore, *MYD88* mutations were associated with an increased risk of developing diffuse large B-cell lymphoma and Hodgkin lymphoma [21,22]. Higher expression of *MYD88* was associated with worse prognosis in colon cancer [23] and has been associated with the incidence of different gynecological cancers, and a potential target therapy role has been evaluated [24]. Although the available data are limited, *TLR4* expression has been examined in head and neck samples in previous studies. A study by Szczepanski et al. evaluated *TLR4* expression and its effects on head and neck carcinomas. *TLR4* was overexpressed in patient samples of laryngeal and oropharyngeal carcinoma and was associated with tumor histological grade (well-differentiated/moderately differentiated tumors had higher *TLR4* expression). The same study examined LPS as a ligand for TLR4 signaling in tumor cells, which had a proliferative effect, stimulated protective cytokine production, and, therefore, lowered cancer cell apoptosis [25,26]. Starska K et al. also found that *TLR4* expression was significantly lower in tumors invading the thyroid cartilage [27].

Evidently, single-nucleotide polymorphisms in *TLR4* and *MYD88* are significantly associated with cancer development. Unfortunately, their role in LSCC is unclear due to inconsistent results. This study aims to evaluate selected SNPs *TLR4* rs7037225, rs11536889, and rs7037117, as well as *MYD88* rs7744 and rs6853, and to determine their roles in disease development, pathological characteristics, and survival.

## 2. Results

A total of 172 patients with histologically confirmed LSCC and 220 healthy individuals in the control group were tested for *TLR4* and *MYD88* polymorphisms. Both groups were matched for sex (only men participated in the study) and age (*p* = 0.055). The median age at diagnosis for the LSCC group was 63 years (range: 42–86), while the control group had a median age of 65 years (range: 44–75). Descriptive characteristics of the study population, along with the data from medical records, are summarized in Table 1.

The frequencies of the relevant genotype variants in the analyzed population were similar to those reported in the European Population Allele Frequencies data from the 1000 Genomes Project Database. *TLR4* rs11536889 and rs7037117, as well as *MYD88* rs7744 and rs6853 genotype frequencies, were in the Hardy–Weinberg equilibrium (HWE). One polymorphism (*TLR4* rs7037225) deviated from the HWE in the control group. Given the satisfactory genotyping quality metrics and the absence of deviation among other analyzed polymorphisms, this variant was retained in the study; however, the results involving this SNP should be interpreted with caution.

The genotype distributions of *TLR4* rs7037225, rs11536889, and rs7037117, as well as *MYD88* rs7744 and rs6853 polymorphisms, in the laryngeal cancer patient and control groups are presented in Table 2. There were no differences in genotype and allele distributions between the control and LSCC groups.

Additionally, the relationships between the selected SNPs and the pathomorphological characteristics of LSCC were analyzed, and the results are summarized in Table 3.

Analysis of *MYD88* rs7744 revealed several significant associations with clinicopathological features of LSCC, including tumor size, lymph node involvement, and clinical stage. Patients with the AG genotype had a significantly lower proportion of large tumors (T3–4) (*p* = 0.027), and this genotype was associated with a protective effect against larger tumor size (OR = 0.429, 95% CI: 0.220–0.837, *p* = 0.013). Similarly, analysis at the allele level showed that *MYD88* rs7744 G allele carriers were significantly less frequent among patients with larger tumors (25.3% in T3–4 vs. 41.6% in T1–2, *p* = 0.023). The G allele was also associated with a reduced risk of developing larger tumors (OR = 0.475, 95% CI: 0.249–0.909, *p* = 0.024), suggesting a tumor size-modifying effect for this variant. Furthermore, patients harboring the AG genotype (vs. the AA genotype) had a significantly lower risk of lymph node involvement (OR = 0.410, 95% CI 0.198–0.848, *p* = 0.016). Similarly, G allele carriers had a significantly lower risk of lymph node involvement (OR = 0.450, 95% CI 0.225–0.903, *p* = 0.025). Lastly, patients with the AG genotype (vs. AA) had a significantly lower risk of III–IV clinical stage (OR = 0.451, 95% CI 0.232–0.878, *p* = 0.019), and G allele carriers (vs non-carriers) had a significantly lower risk of III–IV clinical stage (OR = 0.490, 95% CI 0.256–0.938, *p* = 0.031).

Although the chi-square test revealed a statistically significant association between the *TLR4* rs7037117 genotype and lymph node involvement, logistic regression analysis did not show statistically significant associations.

To explore all potential associations, a multivariable analysis of all polymorphisms was additionally performed to determine whether significant associations remained independent after adjusting for potential confounding clinical variables. The results are provided in Table 4.

*MYD88* rs7744 appears to influence key clinicopathological characteristics. Specifically, LSCC patients carrying the AG genotype or G allele showed significantly reduced odds of having larger tumors (T3–4) and nodal metastasis (N1–3). These findings suggest that certain *MYD88* variants may play a protective role in tumor progression and lymph node involvement, potentially by modulating inflammatory or immune-related pathways involved in cancer development. Advanced clinical stage (III–IV) was less likely among the *MYD88* rs7744 AG (vs. AA) genotype and G allele carriers (vs. non-carriers), further supporting the role of these genetic variants in limiting tumor progression. These associations remained statistically significant even after adjusting for confounders in Model II. Moreover, *TLR4* rs11536889 also appears to affect tumor clinical features. Specifically, LSCC patients carrying the GC genotype showed significantly reduced odds of having larger tumors (T3–4) in Model III. Lastly, clinical stage III–IV was less likely among *TLR4* rs7037117 GG carriers (vs. AA) (Model II).

**Figure 1:**
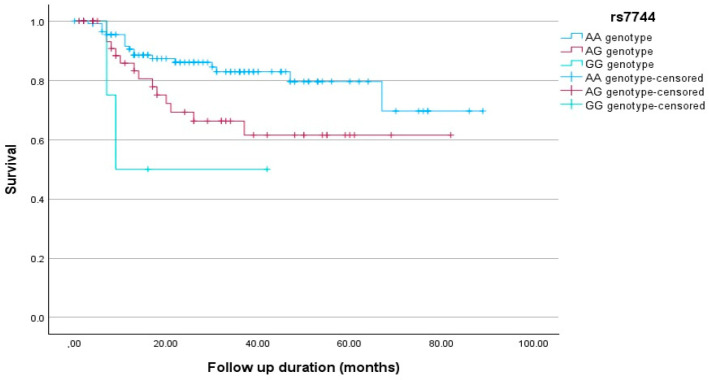
Relapse-free survival rate according to the distribution of *MYD88* rs7744 genotypes.

The present study evaluated overall survival (OS) and relapse-free survival (RFS) in patients with LSCC and examined their associations with *TLR4* and *MYD88* SNPs. *MYD88* rs7744 was significantly associated with RFS, as demonstrated by the log-rank, Breslow, and Tarone–Ware tests (all *p* < 0.05). The results are illustrated in Table 5 and the Kaplan–Meier curves in Figure 1, indicating statistically significant differences in relapse-free survival between the genotypes.

**Figure 2:**
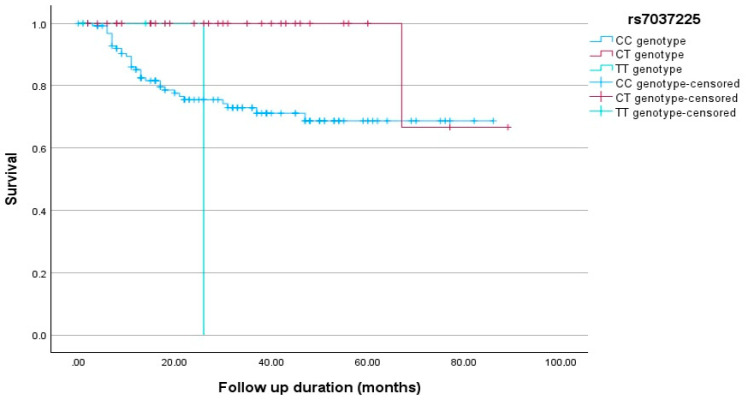
Relapse-free survival rate according to the distribution of *TLR4* rs7037225 genotypes.

Furthermore, we found that *TLR4* rs7037225 was significantly associated with RFS when comparing different genotypes in survival analyses (Figure 2, Table 6).

There were no statistically significant differences in overall survival for the studied SNPs.

Survival analysis was further evaluated using Cox proportional hazards regression (Table 7).

The presence of the AG genotype (vs AA) in *MYD88* rs7744 was significantly associated with poorer recurrence-free survival. The hazard ratios (HRs) ranged from 2.071 to 2.177, with *p* between 0.031 and 0.041 in different models, indicating a consistent, statistically significant association. This suggests that individuals carrying the AG genotype have approximately a two-fold increased risk of disease recurrence compared to those with the AA genotype.

Similarly, the GG genotype (vs. AA) showed a strong trend toward reduced RFS, with HRs ranging from 4.307 to 4.778 and *p* ranging from 0.037 to 0.052 in different models. In most models, this association was statistically significant, suggesting a 4- to 5-fold increased risk of recurrence in GG carriers. However, the wide confidence intervals (e.g., 1.100–20.759) and a borderline *p* in one model (0.052) indicate some uncertainty, possibly due to a smaller number of GG carriers. 

In contrast, carriers of the CT genotype (vs. CC) in *TLR4* rs7037225 demonstrated consistently and significantly better RFS, with hazard ratios ranging from 0.100 to 0.120 and *p* between 0.024 and 0.037 across models. This suggests that the CT genotype is associated with a ~80–90% reduction in the risk of recurrence, indicating a potentially protective effect. However, the TT genotype (vs. CC) was not significantly associated with RFS. The hazard ratios were above 2.0 in all models (suggesting a possible increased risk), but the *p*-values were all non-significant (0.191–0.448), and the confidence intervals were wide, overlapping 1.0. Therefore, no reliable conclusion can be drawn about the effect of the TT genotype.

Full data for the survival analysis are provided in Supplementary Material File S1, “Supplementary Material” (Figures S1–S8, Tables S1–S8).

## 3. Discussion

*TLR4* and *MYD88* single-nucleotide polymorphisms appear to have a significant impact on the pathogenesis of many autoimmune and malignant diseases. TLR signaling can contribute to cancer development through multiple mechanisms and at various stages, including microbial infection (e.g., HPV infection), tissue injury, chronic inflammation, and impaired tissue repair. TLR4 activation also stimulates macrophage activation, leading to the production of inflammatory proteins, such as vascular endothelial growth factor (VEGF), interleukin-6 (IL-6), interleukin-10 (IL-10), and prostaglandins. These factors facilitate tumor invasiveness by promoting angiogenesis, extracellular matrix remodeling, and additional microenvironmental changes [28].

The *TLR4* rs11536889 variant has been examined across various cancer types with mixed results. Castaño-Rodríguez et al. found an association between rs11536889 and increased gastric cancer risk in a Chinese population [29], while Zerrad et al. reported a decreased risk of hepatocellular carcinoma linked to the same variant in a Moroccan cohort [30]. Conversely, Zhang et al. observed an increased risk of hepatocellular carcinoma associated with rs11536889 in a Chinese group [31]. No significant link was found with nasopharyngeal carcinoma in the study by Chaaben et al. [32]. Additionally, a systematic review by Weng et al. did not support a connection between rs11536889 and aggressive prostate cancer [15]. Other studies have shown no associations with acute myeloid leukemia [33] or HPV-related cervical cancer risk [34]. In our study, rs11536889 did not affect disease incidence or survival; however, it demonstrated a protective association with tumor size in the multivariate analysis, suggesting that this SNP may affect tumor growth rather than initiation or long-term outcomes.

The *TLR4* rs7037225 polymorphism has not yet been studied in relation to cancer susceptibility or survival outcomes. In our study, the *TLR4* rs7037225 CT genotype was significantly associated with improved RFS, suggesting a protective effect, while the TT genotype did not show a statistically significant impact. This suggests an advantage of a more balanced heterozygous genotype in TLR4 signaling. Additionally, the low frequency of the TT genotype in the study population may have limited the statistical power to detect its effect.

In general, the *TLR4* rs7037117 polymorphism has not been investigated in the context of cancer but has been studied in non-cancerous diseases. Its role was examined in glaucoma [34,35], autoimmune pancreatitis [36], and Alzheimer’s disease [37], where a possible protective effect was suggested. Our data indicate that carriers of the *TLR4* rs7037117 GG genotype had a lower likelihood of advanced-stage disease than AA carriers in the multivariate analysis, suggesting a potential protective effect. Nonetheless, no previous studies are available for direct comparison.

MYD88 is a crucial adaptor protein in Toll-like receptor signaling, facilitating activation of the NF-κB pathway and subsequent inflammatory responses, which are known to contribute to carcinogenesis and tumor progression. The *MYD88* rs7744 variant has been linked to several autoimmune and inflammatory diseases, such as cervical lesions [38], rheumatoid arthritis [39], and ulcerative colitis [40]. However, evidence of its role in cancer development is limited, and its significance in oncology remains largely unexplored. Our analysis of the *MYD88* rs7744 AG genotype identified several significant links with LSCC clinicopathological features, including tumor size, lymph node involvement, and clinical stage, suggesting a possible protective effect that persisted in the multivariate analysis. In contrast, the *MYD88* rs7744 polymorphism was associated with significantly reduced recurrence-free survival. Both the AG and GG genotypes appeared to be unfavorable prognostic factors, with GG showing the highest risk. These findings indicate that rs7744 might affect tumor characteristics differently at diagnosis and play distinct roles during long-term tumor development. It may be protective in the early stages of disease, although it could later compromise the immune system’s control of tumor progression.

*MYD88* rs6853 has been studied in relation to autoimmune and metabolic disorders, such as rheumatoid arthritis [11] and type II diabetes mellitus [14]. However, there have been no definitive studies examining its role in cancer susceptibility to date, and our study did not find an association with incidence, survival, or clinical features of LSCC.

There was a low incidence of distant metastasis in the LSCC group (four patients, 2.3%). We included this data in our research because it is consistent with other published results, which report distant metastasis rates ranging from 3.21% to 5%. However, this could also be due to difficulties in diagnosing distant metastasis, issues with medical reporting, and loss of patient follow-up [3].

This study faces limitations typical of its retrospective design, such as potential selection bias and reliance on existing medical records, which may lead to incomplete data collection. Another limitation of this research is the absence of environmental factors, especially smoking and alcohol use. However, these were not considered because their effects have been thoroughly studied in previous research [5,41,42].

Despite these limitations, retrospective analyses remain valuable for exploring genetic links in relatively uncommon cancers like LSCC. The study included a sufficiently large sample size (392 participants, including both patients and controls) to detect meaningful associations. Moreover, the inclusion of a homogeneous patient group of 172 LSCC patients from a single cancer center, evaluated using a standardized diagnostic protocol, provides a valuable dataset for future meta-analyses. Since no prior studies have addressed these polymorphisms in relation to LSCC, our findings are novel and provide important new insights into the development of LSCC. Identifying new immunity-related genetic variants could enhance risk stratification in LSCC, especially for individuals exposed to significant environmental risk factors.

## 4. Materials and Methods

### 4.1. Study Design

This retrospective case–control study enrolled 172 men with LSCC, and all adult male patients were treated at the Department of Otorhinolaryngology, Lithuanian University of Health Sciences (LUHS) from 2017 to 2024. Each participant underwent an otorhinolaryngology specialist examination using a video laryngoscope and/or a flexible endoscope in the outpatient clinic. Additionally, radiological assessments included computed tomography and/or magnetic resonance imaging, along with screening for distant metastases. Tumor biopsies were obtained by direct microlaryngoscopy under general anesthesia in the inpatient setting. The Department of Pathology at LUHS conducted histological analyses to confirm LSCC diagnosis and determine tumor differentiation grade (G). Tumor staging and classification were established based on histopathological and radiological findings using the National Comprehensive Cancer Network (NCCN) guidelines (version 2.2020).

Patients younger than 18 years and those with a history of other malignant diseases or inflammatory disorders were excluded from the study. Female patients were not included because, according to the newest data from the World Health Organization Global Cancer Observatory, only 6.3% of laryngeal cancer cases in Lithuania in 2022 occurred in women, resulting in an insufficient number of eligible cases for inclusion in the present study [7].

The control group comprised 220 men who underwent routine annual health examinations by a family medicine specialist at the Department of Family Medicine, LUHS. Individuals younger than 18 years old and those with inflammatory disorders and/or other primary malignancies at the time of the examination were excluded from the study.

Information on tumor size (T), nodal metastasis (N), distant metastasis (M), staging (ST), and histologic grade (G) was collected from medical records. Groups were divided according to tumor size: T1–2 (small/early tumors) and T3–4 (large/locally advanced tumors); lymph node involvement, N0 (no lymph node involvement) and N1–3 (lymph node metastasis detected); distant metastasis, M0 (no distant metastasis) vs. M1 (distant metastasis detected); and histologic differentiation, G1–2 (well-/moderately differentiated tumor) vs. G3–4 (poor/undifferentiated tumor) and stage ST1–2 (early) and ST3–4 (advanced).

### 4.2. Genotyping of Selected Polymorphisms

The polymorphisms *TLR4* rs7037225, rs11536889, and rs7037117, as well as *MYD88* rs7744 and rs6853, were identified via the dbSNP database (https://www.ncbi.nlm.nih.gov/snp/, accessed on 10 September 2025). Polymorphisms with a minor allele frequency (MAF) of at least 5% were selected. Genotyping of the selected polymorphisms was performed at the Oncology Research Laboratory of the Institute of Oncology at the LUHS. Venous blood samples were collected in EDTA-containing vacutainers and stored at −20 °C until further processing. Genomic DNA was extracted from each subject’s peripheral blood leukocytes using a commercially available DNA extraction kit (Thermo Fisher Scientific, Waltham, MA, USA). The assays IDs were C__30249154_10, C__31784034_10, C__29961120_10, C___3094830_10, and C__8824617_10. DNA concentration was determined using the Multiskan SkyHigh microplate spectrophotometer (A51119600DPC, Thermo Fisher Scientific, Waltham, MA, USA). DNA samples were subsequently standardized to a concentration of 10 ng/μL for further analysis. Polymorphisms were evaluated with TaqMan^®^ (Applied Biosystems Europe BV, UK Branch, Warrington, Cheshire, UK) following the manufacturer’s guidelines on the QuantStudio 3 real-time PCR platform (Applied Biosystems, Foster City, CA, USA).

### 4.3. Sample Size

The formula *n* = Z^2^ × p × (1 − p)/E^2^ was applied to calculate the necessary sample size for this study, where n denotes the required sample size, Z is the level of confidence (Z = 1.96 for a 95% confidence level), p is the minimum allele frequency, and E is the acceptable margin of error. The minimal allele frequency was obtained from the SNP database and ranged from 13.12 to 24.35% in the European database (1000 Genome Project). The margin of error was set at 8%. Based on these parameters, the sample size ranged from 69 to 111 participants.

### 4.4. Statistical Analysis

Statistical analysis was conducted using the “Statistical Package for the Social Sciences, version 30.0 for Windows” (SPSS for Windows, version 30.0.0.0, Armonk, New York, NY, USA). The Shapiro–Wilk test was used to assess the normality of the measured characteristics. Since the data did not meet normal distribution criteria, the median was used for descriptive statistics. The Mann–Whitney U test was applied to compare results across groups with non-normal distributions. Genotype distributions were examined for the Hardy–Weinberg equilibrium (HWE). Associations between *TLR4* and *MYD88* gene polymorphisms and clinical or pathological features were analyzed with χ^2^ and Fisher’s exact tests. Binary logistic regression evaluated odds ratios (ORs) with 95% confidence intervals (CIs). To address potential bias, a multivariate logistic regression model estimated adjusted ORs. Results with *p* < 0.05 were considered statistically significant.

In survival analysis, associations between the tested polymorphisms and overall survival (OS) as well as relapse-free survival (RFS) were evaluated. Survival curves were generated using the Kaplan–Meier method and compared using the log-rank, Breslow, and Tarone–Ware tests. Cox regression analysis was used to calculate the hazard ratio (HR) and 95% CI for the association between genotypes and the risk of relapse and death, with adjustments for age at diagnosis, T, N, and G. Statistical significance was defined as a *p* < 0.05.

Cause of death for deceased patients was collected from the Lithuanian State Register of Death Cases and Their Causes, and the overall survival rate was also collected.

## 5. Conclusions

The present study provides a better understanding of the development and progression of LSCC. Several polymorphisms demonstrated potential protective associations with tumor characteristics, including smaller tumor size and lower odds of advanced-stage disease and lymph node metastasis. In survival analysis, the *TLR4* rs7037225 CT genotype (vs. CC) was associated with improved relapse-free survival, whereas the *MYD88* rs7744 AG genotype (vs. AA) was linked to reduced relapse-free survival.

None of the polymorphisms influenced LSCC incidence. Collectively, these findings suggest that *TLR4* and *MYD88* genetic variants may play a role in disease progression and prognosis rather than cancer susceptibility. Future research, including multiple cancer centers with larger sample sizes, is necessary to validate these associations.

## Figures and Tables

**Table 1:** Demographic characteristics of the study group.

Characteristics	Values
Median age at diagnosis, LSCC group (*n* = 172) (years, min–max)	63 (42–86) *
Median age of the control group (*n* = 220) (years, min–max)	65 (44–75) *
T1, *n* (%)	54 (31.4)
T2, *n* (%)	23 (13.3)
T3, *n* (%)	45 (26.2)
T4, *n* (%)	50 (29.1)
T1–2, *n* (%)	77 (44.8)
T3–4, *n* (%)	95 (55.2)
N0, *n* (%)	105 (61.0)
N1, *n* (%)	21 (12.2)
N2, *n* (%)	39 (22.7)
N3, *n* (%)	7 (4.1)
N1–3, *n* (%)	67 (39.0)
M0, *n* (%)	168 (97.7)
M1, *n* (%)	4 (2.3)
G1, *n* (%)	26 (15.3)
G2, *n* (%)	113 (66.5)
G3, *n* (%)	30 (17.6)
G4, *n* (%)	1 (0.6)
G1–2, *n* (%)	139 (81.8)
G3–4 *n* (%)	31 (18.2)
ST I, *n* (%)	51 (29.6)
ST II, *n* (%)	18 (10.5)
ST III, *n* (%)	32 (18.6)
ST IV, *n* (%)	71 (41.3)
ST I–II, *n* (%)	69 (40.1)
ST III–IV, *n* (%)	103 (59.9)

* Groups were matched by age (Mann–Whitney U test, *p* = 0.055). Abbreviations: LSCC, laryngeal squamous cell carcinoma; T1–2, small tumors, T3–4, large tumors; N0, lymph nodes without metastasis, N1–3, lymph nodes with metastasis; M0, no distant metastasis to other organs, M1, distant metastasis to other organs; G1–2, well-/moderately differentiated tumors, G3–4, poorly/undifferentiated tumors; ST I–II, early clinical stage, ST III–IV, advanced clinical stage.

**Table 2:** SNP genotype and allele distribution in the LSCC and control groups.

Gene	Polymorphism	Genotype/Allele	LSCC Cases, *n* (%)	Control, *n* (%)	*p*	OR (95% CI)	*p*
*TLR4*	rs7037225	CC	136 (79.1)	173 (78.6)	0.510	Reference	
		CT	33 (19.2)	39 (17.7)		1.076 (0.643–1.802)	0.780
		TT	3 (1.7)	8 (3.6)		0.477 (0.124–1.832)	0.281
		C allele non-carriers	3 (1.7)	8 (3.6)	0.360	Reference	
		C allele carriers	169 (98.3)	212 (96.4)		2.126 (0.555–8.136)	0.271
		T allele non-carriers	136 (79.1)	173 (78.6)	0.917	Reference	
		T allele carriers	36 (20.9)	47 (21.4)		0.974 (0.598–1.588)	0.917
*TLR4*	rs11536889	GG	139 (80.8)	182 (82.7)	0.628	Reference	
		GC	32 (18.6)	35 (15.9)		1.197 (0.706–2.029)	0.504
		CC	1 (0.6)	3 (1.4)		0.436 (0.045–4.241)	0.475
		G allele non-carriers	1 (0.6)	3 (1.4)	0.634	Reference	
		G allele carriers	171 (99.4)	217 (98.6)		2.364 (0.244–22.929)	0.458
		C allele non-carriers	139 (80.8)	182 (82.7)	0.625	Reference	
		C allele carriers	33 (19.2)	38 (17.3)		1.137 (0.679–1.905)	0.626
*TLR4*	rs7037117	AA	110 (64.0)	133 (60.5)	0.651	Reference	
		AG	52 (30.2)	76 (34.5)		0.827 (0.536–1.277)	0.827
		GG	10 (5.8)	11 (5.0)		1.099 (0.450–2.684)	0.836
		A allele non-carriers	10 (5.8)	11 (5.0)	0.722	Reference	
		A allele carriers	162 (94.2)	209 (95.0)		0.853 (0.353–2.057)	0.723
		G allele non-carriers	110 (64.0)	133 (60.5)	0.479	Reference	
		G allele carriers	62 (36.0)	87 (39.5)		0.862 (0.571–1.301)	0.479
*MYD88*	rs7744	AA	116 (67.4)	136 (61.8)	0.340	Reference	
		AG	52 (30.2)	74 (33.6)		0.824 (0.535–1.270)	0.380
		GG	4 (2.3)	10 (4.5)		0.469 (0.143–1.535)	0.211
		A allele non-carriers	4 (2.3)	10 (4.5)	0.240	Reference	
		A allele carriers	168 (97.7)	210 (95.5)		2.000 (0.616–6.490)	0.248
		G allele non-carriers	116 (67.4)	136 (61.8)	0.249	Reference	
		G allele carriers	56 (32.6)	84 (38.2)		0.782 (0.514–1.189)	0.249
*MYD88*	rs6853	AA	127 (73.8)	164 (74.5)	0.964	Reference	
		AG	44 (25.6)	54 (24.5)		1.052 (0.664–1.668)	0.829
		GG	1 (0.6)	2 (0.9)		0.646 (0.058–7.200)	0.722
		A allele non-carriers	1 (0.6)	2 (0.9)	1.000	Reference	
		A allele carriers	171 (99.4)	218 (99.1)		1.569 (0.141–17.446)	0.714
		G allele non-carriers	127 (73.8)	164 (74.5)	0.874	Reference	
		G allele carriers	45 (26.2)	56 (25.5)		1.038 (0.658–1.637)	0.874

Abbreviations: OR, odds ratio; CI, confidence interval.

**Table 3:** Analysis of polymorphism association with the pathomorphological LSCC characteristics.

Polymorphism/Pathomorphological Characteristics		Frequency, *n* (%)		*p* *	OR (95% CI)	*p*
Tumor size (T)		*T1–2*	*T3–4*			
*TLR4* rs7037225	CC	61 (79.2)	75 (78.9)	0.143	Reference	
	CT	13 (16.9)	20 (21.1)		1.251 (0.576–2.718)	0.571
	TT	3 (3.9)	0 (0)		x	x
	C allele non-carriers	3 (3.9)	0 (0)	0.088	Reference	
	C allele carriers	74 (96.1)	95 (100)		x	x
	T allele non-carriers	61 (79.2)	75 (78.9)	0.965	Reference	
	T allele carriers	16 (20.8)	20 (21.10		1.017 (0.485–2.129)	0.965
*TLR4* rs11536889	GG	58 (75.3)	81 (85.3)	0.075	Reference	
	GC	19 (24.7)	13 (13.6)		0.490 (0.224–1.071)	0.074
	CC	0 (0)	1 (1.1)		x	x
	G allele non-carriers	0 (0)	1 (1.1)	1.000	Reference	
	G allele carriers	77 (100)	94 (98.9)		x	x
	C allele non-carriers	58 (75.3)	81 (85.3)	0.100	Reference	
	C allele carriers	19 (24.7)	14 (14.7)		0.528 (0.245–1.138)	0.103
*TLR4* rs7037117	AA	47 (61.0)	63 (66.3)	0.251	Reference	
	AG	23 (29.9)	29 (30.5)		0.941 (0.484–1.829)	0.857
	GG	7 (9.1)	3 (3.2)		0.320 (0.079–1.302)	0.111
	A allele non-carriers	7 (9.1)	3 (3.2)	0.114	Reference	
	A allele carriers	70 (90.9)	92 (96.8)		3.067 (0.766–12.285)	0.114
	G allele non-carriers	47 (61.0)	63 (66.3)	0.474	Reference	
	G allele carriers	30 (39.0)	32 (33.7)		0.796 (0.426–1.487)	0.474
*MYD88* rs7744	AA	45 (58.4)	71 (74.7)	0.027	Reference	
	AG	31 (40.3)	21 (22.1)		0.429 (0.220–0.837)	0.013
	GG	1 (1.3)	3 (3.2)		1.901 (0.192–18.847)	0.583
	A allele non-carriers	1 (1.3)	3 (3.2)	0.629	Reference	
	A allele carriers	76 (98.7)	92 (96.8)		0.404 (0.041–3.959)	0.436
	G allele non-carriers	45 (58.4)	71 (74.7)	0.023	Reference	
	G allele carriers	32 (41.6)	24 (25.3)		0.475 (0.249–0.909)	0.024
*MYD88* rs6853	AA	57 (74.0)	70 (73.7)	1.000	Reference	
	AG	20 (26.0)	24 (25.2)		0.977 (0.491–1.946)	0.948
	GG	0 (0)	1 (1.1)		x	x
	A allele non-carriers	0 (0)	1 (1.1)	1.000	Reference	
	A allele carriers	77 (100)	94 (98.9)		x	x
	G allele non-carriers	57 (74.0)	70 (73.7)	0.960	Reference	
	G allele carriers	20 (26.0)	25 (26.3)		1.018 (0.514–2.017)	0.960
Lymph node involvement (N)		*N0*	*N1–3*			
*TLR4* rs7037225	CC	84 (80.0)	52 (77.6)	0.344	Reference	
	CT	18 (17.1)	15 (22.4)		1.346 (0.625–2.900)	0.448
	TT	3 (2.9)	0 (0)		x	x
	C allele non-carriers	3 (2.9)	0 (0)	0.282	Reference	
	C allele carriers	102 (97.1)	67 (100)		x	x
	T allele non-carriers	84 (80.0)	52 (77.6)	0.707	Reference	
	T allele carriers	21 (20.0)	15 (22.4)		1.154 (0.546–2.436)	0.707
*TLR4* rs11536889	GG	85 (81.0)	54 (80.6)	0.646	Reference	
	GC	20 (19.0)	12 (17.9)		0.944 (0.427–2.087)	0.888
	CC	0 (0)	1 (1.5)		x	x
	G allele non-carriers	0 (0)	1 (1.5)	0.390	Reference	
	G allele carriers	105 (100)	66 (98.5)		x	x
	C allele non-carriers	85 (81.0)	54 (80.6)	1.000	Reference	
	C allele carriers	20 (19.0)	13 (19.4)		1.023 (0.470–2.226)	0.954
*TLR4* rs7037117	AA	67 (63.8)	43 (64.2)	0.023	Reference	
	AG	28 (26.7)	24 (35.8)		1.336 (0.686–2.600)	0.395
	GG	10 (9.5)	0 (0)		x	x
	A allele non-carriers	10 (9.5)	0 (0)	0.007	Reference	
	A allele carriers	95 (90.5)	67 (100)		x	x
	G allele non-carriers	67 (63.8)	43 (64.2)	0.961	Reference	
	G allele carriers	38 (36.2)	24 (35.8)		0.984 (0.520–1.864)	0.961
*MYD88* rs7744	AA	64 (61.0)	52 (77.6)	0.033	Reference	
	AG	39 (37.1)	13 (19.4)		0.410 (0.198–0.848)	0.016
	GG	2 (1.9)	2 (3.0)		1.231 (0.168–9.038)	0.838
	A allele non-carriers	2 (1.9)	2 (3.0)	0.643	Reference	
	A allele carriers	103 (98.1)	65 (97.0)		0.631 (0.087–4.591)	0.649
	G allele non-carriers	64 (61.0)	52 (77.6)	0.023	Reference	
	G allele carriers	41 (39.0)	15 (22.4)		0.450 (0.225–0.903)	0.025
*MYD88* rs6853	AA	78 (74.3)	49 (73.1)	0.614	Reference	
	AG	27 (25.7)	17 (25.4)		1.002 (0.496–2.027)	0.995
	GG	0 (0)	1 (1.5)		x	x
	A allele non-carriers	0 (0)	1 (1.5)	0.390	Reference	
	A allele carriers	105 (100)	66 (98.5)		x	x
	G allele non-carriers	78 (74.3)	49 (73.1)	0.867	Reference	
	G allele carriers	27 (25.7)	18 (26.9)		1.061 (0.530–2.127)	0.867
Metastasis (M)		*M0*	*M1*			
*TLR4* rs7037225	CC	132 (78.6)	4 (100)	1.000	Reference	
	CT	33 (19.6)	0 (0)		x	x
	TT	3 (1.8)	0 (0)		x	x
	C allele non-carriers	3 (1.8)	0 (0)	1.000	Reference	
	C allele carriers	165 (98.2)	4 (100)		x	x
	T allele non-carriers	132 (78.6)	4 (100)	0.581	Reference	
	T allele carriers	36 (21.4)	0 (0)		x	x
*TLR4* rs11536889	GG	135 (80.4)	4 (100)	1.000	Reference	
	GC	32 (19.0)	0 (0)		x	x
	CC	1 (0.6)	0 (0)		x	x
	G allele non-carriers	1 (0.6)	0 (0)	1.000	Reference	
	G allele carriers	167 (99.4)	4 (100)		x	x
	C allele non-carriers	135 (80.4)	4 (100)	1.000	Reference	
	C allele carriers	33 (19.6)	0 (0)		x	x
*TLR4* rs7037117	AA	107 (63.6)	3 (75.0)	1.000	Reference	
	AG	51 (30.4)	1 (25.0)		0.699 (0.071–6.890)	0.759
	GG	10 (6.0)	0 (0)		x	x
	A allele non-carriers	10 (6.0)	0 (0)	1.000	Reference	
	A allele carriers	158 (94.0)	4 (100)		x	x
	G allele non-carriers	107 (63.6)	3 (75.0)	1.000	Reference	
	G allele carriers	61 (36.4)	1 (25.0)		0.585 (0.060–5.745)	0.645
*MYD88* rs7744	AA	112 (66.7)	4 (100)	0.375	Reference	
	AG	52 (31.0)	0 (0)		x	x
	GG	4 (2.3)	0 (0)		x	x
	A allele non-carriers	4 (2.4)	4 (100)	1.000	Reference	
	A allele carriers	164 (97.6)	0 (0)		x	x
	G allele non-carriers	112 (66.7)	4 (100)	0.305	Reference	
	G allele carriers	56 (33.3)	0 (0)		x	x
*MYD88* rs6853	AA	123 (73.2)	4 (100)	0.586	Reference	
	AG	44 (26.2)	0 (0)		x	x
	GG	1 (0.6)	0 (0)		x	x
	A allele non-carriers	1 (0.6)	0 (0)	1.000	Reference	
	A allele carriers	167 (99.4)	4 (100)		x	x
	G allele non-carriers	123 (73.2)	4 (100)	0.574	Reference	
	G allele carriers	45 (26.8)	0 (0)		x	x
Histological grade (G)		*G1–2*	*G3–4*			
*TLR4* rs7037225	CC	110 (79.1)	24 (77.4)	0.706	Reference	
	CT	27 (19.4)	6 (19.4)		1.019 (0.379–2.737)	0.971
	TT	2 (1.4)	1 (3.2)		2.292 (0.200–26.312)	0.505
	C allele non-carriers	2 (1.4)	1 (3.2)	0.456	Reference	
	C allele carriers	137 (98.6)	30 (96.8)		0.438 (0.038–4.988)	0.506
	T allele non-carriers	110 (79.1)	24 (77.4)	0.832	Reference	
	T allele carriers	29 (20.9)	7 (22.6)		1.106 (0.434–2.821)	0.832
*TLR4* rs11536889	GG	110 (79.2)	27 (87.1)	0.557	Reference	
	GC	28 (20.1)	4 (12.9)		0.582 (0.188–1.800)	0.347
	CC	1 (0.7)	0 (0)		x	x
	G allele non-carriers	1 (0.7)	0 (0)	1.000	Reference	
	G allele carriers	138 (99.3)	31 (100)		x	x
	C allele non-carriers	110 (79.2)	27 (87.1)	0.311	Reference	
	C allele carriers	29 (20.8)	4 (12.9)		0.562 (0.182–1.734)	0.316
*TLR4* rs7037117	AA	87 (62.6)	21 (67.7)	0.401	Reference	
	AG	45 (32.4)	7 (22.6)		0.644 (0.255–1.630)	0.353
	GG	7 (5.0)	3 (9.7)		1.776 (0.423–7.449)	0.433
	A allele non-carriers	7 (5.0)	3 (9.7)	0.392	Reference	
	A allele carriers	132 (95.0)	28 (90.3)		0.495 (0.121–2.033)	0.329
	G allele non-carriers	87 (62.6)	21 (67.7)	0.590	Reference	
	G allele carriers	52 (37.4)	10 (32.3)		0.797 (0.348–1.823)	0.590
*MYD88* rs7744	AA	95 (68.3)	19 (61.3)	0.490	Reference	
	AG	40 (28.8)	12 (38.7)		1.500 (0.666–3.377)	0.328
	GG	4 (2.9)	0 (0)		x	x
	A allele non-carriers	4 (2.9)	0 (0)	1.000	Reference	
	A allele carriers	135 (97.1)	31 (100)		x	
	G allele non-carriers	95 (68.3)	19 (61.3)	0.450	Reference	
	G allele carriers	44 (31.7)	12 (38.7)		1.364 (0.609–3.054)	0.451
*MYD88* rs6853	AA	101 (72.7)	25 (80.6)	0.595	Reference	
	AG	37 (26.6)	6 (19.4)		0.655 (0.249–1.724)	0.392
	GG	1 (0.7)	0 (0)		x	x
	A allele non-carriers	1 (0.7)	0 (0)	1.000	Reference	
	A allele carriers	138 (99.3)	31 (100)		x	x
	G allele non-carriers	101 (72.7)	25 (80.6)	0.359	Reference	
	G allele carriers	38 (27.3)	6 (19.4)		0.638 (0.243–1.676)	0.362
Clinical stage (ST)		*ST I–II*	*ST III–IV*			
*TLR4* rs7037225	CC	54 (78.3)	82 (79.6)	0.119	Reference	
	CT	12 (17.4)	21 (20.4)		1.152 (0.524–2.534)	0.724
	TT	3 (4.3)	0 (0)		x	x
	C allele non-carriers	3 (4.3)	0 (0)	0.063	Reference	
	C allele carriers	66 (95.7)	103 (100)		x	x
	T allele non-carriers	54 (78.3)	82 (79.6)	0.831	Reference	
	T allele carriers	15 (21.7)	21 (20.4)		0.922 (0.437–1.945)	0.831
*TLR4* rs11536889	GG	53 (76.8)	86 (83.5)	0.310	Reference	
	GC	16 (23.2)	16 (15.5)		0.616 (0.285–1.335)	0.220
	CC	0 (0)	1 (1.0)		x	x
	G allele non-carriers	0 (0)	1 (1.0)	1.000	Reference	
	G allele carriers	69 (100)	102 (99.0)		x	x
	C allele non-carriers	53 (76.8)	86 (83.5)	0.275	Reference	
	C allele carriers	16 (23.2)	17 (16.5)		0.655 (0.305–1.405)	0.277
*TLR4* rs7037117	AA	42 (60.9)	68 (66.0)	0.139	Reference	
	AG	20 (29.0)	32 (31.1)		0.988 (0.502–1.947)	0.973
	GG	7 (10.1)	3 (2.9)		0.265 (0.650–1.080)	0.064
	A allele non-carriers	7 (10.1)	3 (2.9)	0.091	Reference	
	A allele carriers	62 (89.9)	100 (97.1)		3.763 (0.938–15.096)	0.061
	G allele non-carriers	42 (60.9)	68 (66.0)	0.491	Reference	
	G allele carriers	27 (39.1)	35 (34.0)		0.801 (0.425–1.507)	0.491
*MYD88* rs7744	AA	40 (58.0)	76 (73.8)	0.044	Reference	
	AG	28 (40.6)	24 (23.3)		0.451 (0.232–0.878)	0.019
	GG	1 (1.4)	3 (2.9)		1.579 (0.159–15.675)	0.697
	A allele non-carriers	1 (1.4)	3 (2.9)	0.650	Reference	
	A allele carriers	68 (98.6)	100 (97.1)		0.490 (0.050–4.812)	0.541
	G allele non-carriers	40 (58.0)	76 (73.8)	0.030	Reference	
	G allele carriers	29 (42.0)	27 (26.2)		0.490 (0.256–0.938)	0.031
*MYD88* rs6853	AA	52 (75.4)	75 (72.8)	0.920	Reference	
	AG	17 (24.6)	27 (26.2)		1.101 (0.546–2.223)	0.788
	GG	0 (0)	1 (1.0)		x	x
	A allele non-carriers	0 (0)	1 (1.0)	1.000	Reference	
	A allele carriers	69 (100)	102 (99.0)		x	x
	G allele non-carriers	52 (75.4)	75 (72.8)	0.710	Reference	
	G allele carriers	17 (24.6)	28 (27.2)		1.142 (0.568–2.297)	0.710

*, chi-square (x^2^) test or Fisher’s exact test; OR, odds ratio; CI, confidence interval; x, not applicable.

**Table 4:** Multivariate logistic regression analysis.

Characteristics	Covariates		Model I			Model II			Model III		
			OR	95% CI	*p*	OR	95% CI	*p*	OR	95% CI	*p*
Tumor size T3–4 vs. T1–2	*MYD88* rs7744	AG vs. AA	0.422	0.215–0.827	0.012	0.577	0.268–1.240	0.159	0.530	0.242–1.159	0.112
		GG vs. AA	2.028	0.201–20.413	0.549	1.840	0.142–23.805	0.641	1.794	0.137–23.485	0.656
	Age at diagnosis		1.008	0.975–1.043	0.636	0.992	0.954–1.031	0.672	0.988	0.949–1.027	0.531
	N1–3 vs. N0					11.310	4.962–25.779	0.001	12.158	5.135–28.787	0.001
	G1–3 vs. G0								1.626	0.617–4.286	0.326
	*MYD88* rs7744	A allele carriers vs. A allele non-carriers	0.391	0.039–3.905	0.424	0.465	0.036–6.009	0.558	0.468	0.036–6.128	0.563
	Age at diagnosis		1.004	0.971–1.037	0.830	0.989	0.952–1.027	0.565	0.985	0.947–1.024	0.436
	N1–3 vs. N0					12.159	5.370–27.527	0.001	13.275	5.652–31.182	0.001
	G1–3 vs. G0								1.537	0.586–4.028	0.382
	*MYD88* rs7744	G allele carriers vs. G allele non-carriers	0.473	0.247–0.905	0.024	0.624	0.297–1.311	0.213	0.578	0.271–1.231	0.155
	Age at diagnosis		1.004	0.971–1.038	0.802	0.989	0.952–1.027	0.559	0.985	0.947–1.023	0.429
	N1–3 vs. N0					11.540	5.072–26.255	0.001	12.481	5.284–29.482	0.001
	G1–3 vs. G0								1.571	0.597–4.129	0.360
N1–3 vs. N0	*MYD88* rs7744	AG vs. AA	0.377	0.179–0.792	0.010	0.506	0.217–1.177	0.114	0.501	0.211–1.193	0.118
		GG vs. AA	1.585	0.210–11.935	0.655	1.198	0.125–11.439	0.875	1.364	0.140–13.256	0.789
	Age at diagnosis		1.032	0.996–1.069	0.082	1.036	0.995–1.079	0.086	1.038	0.996–1.082	0.076
	T3–4 vs. T1–2					11.331	4.962–25.879	0.001	12.223	5.146–29.034	0.001
	G1–3 vs. G0								1.626	0.636–4.156	0.310
	*MYD88* rs7744	A allele carriers vs. A allele non-carriers	0.507	0.068–3.785	0.508	0.724	0.076–6.910	0.779	0.644	0.066–6.274	0.705
	Age at diagnosis		1.025	0.991–1.061	0.148	1.032	0.992–1.074	0.116	1.035	0.993–1.077	0.101
	T3–4 vs. T1–2					12.252	5.393–27.833	0.001	13.435	5.691–31.717	0.001
	G1–3 vs. G0								1.509	0.600–3.791	0.382
	*MYD88* rs7744	G allele carriers vs. G allele non-carriers	0.428	0.211–0.867	0.018	0.533	0.247–1.236	0.149	0.559	0.246–1.271	0.165
	Age at diagnosis		1.028	0.993–1.064	0.121	1.033	0.993–1.075	0.104	1.035	0.994–1.078	0.096
	T3–4 vs. T1–2					11.550	5.068–26.324	0.00	12.531	5.289–29.690	0.001
	G1–3 vs. G0								1.558	0.614–3.953	0.351
Clinical stage III–IV vs. I–II	*MYD88* rs7744	AG vs. AA	0.444	0.227–0.868	0.018	0.416	0.210–0.824	0.012			
		GG vs. AA	1.676	0.166–16.905	0.661	1.815	0.179–18.367	0.614			
	Age at diagnosis		1.008	0.974–1.043	0.661	1.006	0.972–1.041	0.742			
	G1–3 vs. G0					2.030	0.848–4.858	0.112			
	*MYD88* rs7744	A allele carriers vs. A allele non-carriers	0.476	0.048–4.759	0.528	0.436	0.043–4.370	0.480			
	Age at diagnosis		1.003	0.970–1.037	0.843	1.002	0.969–1.036	0.913			
	G1–3 vs. G0					1.835	0.785–4.290	0.161			
	*MYD88* rs7744	G allele carriers vs. G allele non-carriers	0.487	0.254–0.934	0.030	0.463	0.239–0.897	0.023			
	Age at diagnosis		1.004	0.971–1.039	0.802	1.002	0.969–1.037	0.899			
	G1–3 vs. G0					1.935	0.814–4.598	0.135			
Tumor size T3–4 vs. T1–2	*MYD88* rs6853	AG vs. AA	0.978	0.491–1.950	0.950	0.954	0.429–2.119	0.907	1.049	0.468–2.350	0.908
		GG vs. AA	x	x	x	x	x	x	x	x	x
	Age at diagnosis		1.001	0.969–1.034	0.963	0.987	0.951–1.025	0.490	0.983	0.946–1.021	0.377
	N1–3 vs. N0					12.016	5.307–27.205	0.001	13.117	5.584–30.812	0.001
	G1–3 vs. G0								1.524	0.583–3.983	0.390
	*MYD88 rs6853*	A allele carriers vs. A allele non-carriers	x	x	x	x	x	x	x	x	x
	Age at diagnosis		1.001	0.969–1.034	0.959	0.987	0.951–1.025	0.494	0.983	0.946–1.021	0.374
	N1–3 vs. N0					12.012	5.306–27.195	0.001	13.113	5.583–30.800	0.001
	G1–3 vs. G0								1.524	0.583–3.987	0.390
	*MYD88 rs6853*	G allele carriers vs. G allele non-carriers	1.020	0.514–2.024	0.955	0.971	0.439–2.150	0.942	1.067	0.478–2.384	0.874
	Age at diagnosis		1.002	0.970–1.035	0.917	0.987	0.951–1.025	0.500	0.983	0.946–1.022	0.386
	N1–3 vs. N0					12.232	5.405–27.686	0.001	13.370	5.695–31.392	0.001
	G1–3 vs. G0								1.513	0.579–3.958	0.398
N1–3 vs. N0	*MYD88* rs6853	AG vs. AA	1.305	0.510–2.103	0.924	1.063	0.467–2.420	0.885	0.972	0.420–2.251	0.947
		GG vs. AA	x	x	x	x	x	x	x	x	x
	Age at diagnosis		1.023	0.989–1.058	0.191	1.031	0.991–1.072	0.133	1.032	0.991–1.075	0.123
	T3–4 vs. T1–2					12.095	5.326–27.467	0.001	13.254	5.617–31.276	0.001
	G1–3 vs. G0								1.510	0.601–3.796	0.381
	*MYD88* rs6853	A allele carriers vs. A allele non-carriers	x	x	x	x	x	x	x	x	x
	Age at diagnosis		1.023	0.989–1.057	0.192	1.030	0.991–1.072	0.135	1.032	0.992–1.075	0.122
	T3–4 vs. T1–2					12.090	5.324–27.453	0.001	13.251	5.616–31.265	0.001
	G1–3 vs. G0								1.514	0.604–3.792	0.376
	*MYD88* rs6853	G allele carriers vs. G allele non-carriers	1.094	0.543–2.203	0.801	1.110	0.491–2.509	0.801	1.020	0.444–2.342	0.963
	Age at diagnosis		1.024	0.990–1.059	0.167	1.032	0.992–1.073	0.120	1.033	0.993–1.076	0.109
	T3–4 vs. T1–2					12.314	5.424–27.956	0.001	13.519	5.731–31.891	0.001
	G1–3 vs. G0								1.491	0.593–3.751	0.396
Tumor size T3–4 vs. T1–2	*TLR4* rs7037225	CT vs. CC	1.247	0.573–2.715	0.578	1.128	0.459–2.773	0.793	1.056	0.426–2.620	0.906
		TT vs. CC	x	x	x	x	x	x	x	x	x
	Age at diagnosis		1.002	0.970–1.035	0.908	0.988	0.952–1.026	0.530	0.984	0.947–1.022	0.412
	N1–3 vs. N0					11.611	5.122–26.318	0.001	12.646	5.374–29.755	0.001
	G1–3 vs. G0								1.604	0.600–4.292	0.346
	*TLR4* rs7037225	C allele carriers vs. C allele non-carriers	x	x	x	x	x	x	x	x	x
	Age at diagnosis		1.003	0.970–1.036	0.875	0.988	0.952–1.026	0.541	0.984	0.948–1.022	0.415
	N1–3 vs. N0					11.656	5.144–26.412	0.001	12.676	5.392–29.804	0.001
	G1–3 vs. G0								1.608	0.601–4.302	0.344
	*TLR4* rs7037225	T allele carriers vs. T allele non-carriers	1.014	0.483–2.128	0.971	0.949	0.400–2.254	0.906	0.885	0.369–2.123	0.784
	Age at diagnosis		1.002	0.969–1.035	0.922	0.987	0.951–1.025	0.509	0.984	0.947–1.022	0.395
	N1–3 vs. N0					12.241	5.407–27.711	0.001	13.429	5.714–31.559	0.001
	G1–3 vs. G0								1.525	0.580–4.011	0.392
N1–3 vs. N0	*TLR4* rs7037225	CT vs. CC	1.297	0.599–2.810	0.510	1.206	0.492–2.955	0.682	1.254	0.507–3.099	0.624
		TT vs. CC	x	x	x	x	x	x	x	x	x
	Age at diagnosis		1.023	0.990–1.058	0.175	1.031	0.991–1.072	0.131	1.032	0.992–1.075	0.121
	T3–4 vs. T1–2					11.691	5.141–26.589	0.001	12.786	5.408–30.229	0.001
	G1–3 vs. G0								1.528	0.607–3.846	0.369
	*TLR4* rs7037225	C allele carriers vs. C allele non-carriers	x	x	x	x	x	x	x	x	x
	Age at diagnosis		1.024	0.991–1.059	0.160	1.031	0.992–1.073	0.120	1.033	0.993–1.076	0.108
	T3–4 vs. T1–2					11.746	5.166–26.708	0.001	12.830	5.428–30.324	0.001
	G1–3 vs. G0								1.521	0.603–3.838	0.375
	*TLR4* rs7037225	T allele carriers vs. T allele non-carriers	1.109	0.522–2.355	0.788	1.129	0.468–2.723	0.787	1.173	0.482–2.855	0.726
	Age at diagnosis		1.023	0.990–1.058	0.179	1.031	0.991–1.072	0.130	1.033	0.991–1.075	0.119
	T3–4 vs. T1–2					12.316	5.425–27.964	0.001	13.559	5.745–32.002	0.001
	G1–3 vs. G0								1.490	0.596–3.726	0.394
Tumor size T3–4 vs. T1–2	*TLR4* rs11536889	GC vs. GG	0.489	0.224–1.069	0.073	0.391	0.152–1.007	0.052	0.372	0.141–0.981	0.046
		CC vs. GG	x	x	x	x	x	x	x	x	x
	Age at diagnosis		1.002	0.970–1.036	0.900	0.989	0.952–1.027	0.553	0.985	0.948–1.024	0.441
	N1–3 vs. N0					12.780	5.528–29.548	0.001	14.203	5.890–34.248	0.001
	G1–3 vs. G0								1.414	0.533–3.752	0.486
	*TLR4 rs11536889*	G allele carriers vs. G allele non-carriers	x	x	x	x	x	x	x	x	x
	Age at diagnosis		1.001	0.969–1.034	0.959	0.987	0.951–1.025	0.494	0.983	0.946–1.021	0.374
	N1–3 vs. N0					12.012	5.306–27.195	0.001	13.113	5.583–30.800	0.001
	G1–3 vs. G0								1.524	0.583–3.987	0.390
	*TLR4 rs11536889*	C allele carriers vs. C allele non-carriers	0.526	0.244–1.135	0.101	0.407	0.159–1.038	0.060	0.387	0.148–1.010	0.052
	Age at diagnosis		1.003	0.971–1.037	0.855	0.989	0.952–1.027	0.568	0.985	0.948–1.024	0.454
	N1–3 vs. N0					13.107	5.666–30.319	0.001	14.610	6.053–35.260	0.001
	G1–3 vs. G0								1.400	0.527–3.721	0.499
N1–3 vs. N0	*TLR4* rs11536889	GC vs. GG	0.924	0.416–2.051	0.846	1.539	0.581–4.075	0.385	1.682	0.620–4.558	0.307
		CC vs. GG	x	x	x	x	x	x	x	x	x
	Age at diagnosis		1.023	0.989–1.058	0.190	1.030	0.990–1.071	0.144	1.031	0.990–1.074	0.136
	T3–4 vs. T1–2					12.878	5.550–29.881	0.001	14.358	5.927–34.787	0.001
	G1–3 vs. G0								1.551	0.617–3.901	0.351
	*TLR4* rs11536889	G allele carriers vs. G allele non-carriers	x	x	x	x	x	x	x	x	x
	Age at diagnosis		1.023	0.989–1.057	0.192	1.030	0.991–1.072	0.135	1.032	0.992–1.075	0.122
	T3–4 vs. T1–2					12.090	5.324–27.453	0.001	13.251	5.616–31.265	0.001
	G1–3 vs. G0								1.514	0.604–3.792	0.376
	*TLR4* rs11536889	C allele carriers vs. C allele non-carriers	0.997	0.456–2.179	0.993	1.626	0.622–4.248	0.321	1.780	0.665–4.762	0.251
	Age at diagnosis		1.024	0.990–1.059	0.171	1.031	0.991–1.072	0.135	1.032	0.991–1.075	0.127
	T3–4 vs. T1–2					13.192	5.685–30.613	0.001	14.746	6.085–35.735	0.001
	G1–3 vs. G0								1.536	0.610–3.868	0.363
Tumor size T3–4 vs. T1–2	*TLR4* rs7037117	AG vs. AA	0.940	0.483–1.828	0.855	0.753	0.343–1.654	0.480	0.707	0.317–1.574	0.396
		GG vs. AA	0.320	0.079–1.302	0.122	0.715	0.170–3.000	0.646	0.639	0.150–2.733	0.546
	Age at diagnosis		1.002	0.969–1.035	0.924	0.988	0.951–1.025	0.511	0.983	0.947–1.002	0.392
	N1–3 vs. N0					12.268	5.329–28.243	0.001	13.477	5.619–32.326	0.001
	G1–3 vs. G0								1.539	0.576–4.116	0.390
	*TLR4* rs7037117	A allele carriers vs. A allele non-carriers	3.066	0.765–12.281	0.114	1.291	0.313–5.328	0.724	1.419	0.337–5.965	0.633
	Age at diagnosis		1.002	0.969–1.035	0.927	0.987	0.951–1.025	0.506	0.983	0.946–1.021	0.384
	N1–3 vs. N0					11.940	5.225–27.285	0.001	12.928	5.451–30.663	0.001
	G1–3 vs. G0								1.561	0.589–4.138	0.371
	*TLR4* rs7037117	G allele carriers vs. G allele non-carriers	0.795	0.426–1.486	0.473	0.746	0.359–1.547	0.430	0.693	0.330–1.454	0.332
	Age at diagnosis		1.002	0.970–1.035	0.909	0.988	0.951–1.025	0.511	0.983	0.947–1.022	0.392
	N1–3 vs. N0					12.335	5.436–27.987	0.001	13.629	5.777–32.154	0.001
	G1–3 vs. G0								1.526	0.576–4.042	0.395
N1–3 vs. N0	*TLR4* rs7037117	AG vs. AA	1.327	0.679–2.593	0.408	1.525	0.691–3.364	0.296	1.673	0.745–3.760	0.213
		GG vs. AA	x	x	x	x	x	x	x	x	x
	Age at diagnosis		1.024	0.990–1.059	0.174	1.031	0.991–1.072	0.135	1.032	0.991–1.075	0.131
	T3–4 vs. T1–2					12.296	5.329–28.370	0.001	13.517	5.613–32.553	0.001
	G1–3 vs. G0								1.761	0.669–4.638	0.252
	*TLR4* rs7037117	A allele carriers vs. A allele non-carriers	x	x	x	x	x	x	x	x	x
	Age at diagnosis		1.024	0.990–1.060	0.170	1.031	0.991–1.073	0.127	1.033	0.992–1.076	0.116
	T3–4 vs. T1–2					12.004	5.235–27.523	0.001	13.036	5.467–31.081	0.001
	G1–3 vs. G0								1.678	0.639–4.408	0.294
	*TLR4* rs7037117	G allele carriers vs. G allele non-carriers	0.977	0.514–1.856	0.942	1.129	0.533–2.393	0.751	1.208	0.562–2.596	0.628
	Age at diagnosis		1.024	0.990–1.058	0.171	1.031	0.992–1.073	0.125	1.033	0.992–1.075	0.114
	T3–4 vs. T1–2					12.404	5.453–28.220	0.001	13.727	5.797–32.506	0.001
	G1–3 vs. G0								1.504	0.601–3.768	0.383
Clinical stage III–IV vs. I–II	*TLR4* rs7037117	AG vs. AA	0.988	0.501–1.946	0.971	1.017	0.512–2.021	0.961			
		GG vs. AA	0.265	0.065–1.080	0.064	0.237	0.057–0.993	0.049			
	Age at diagnosis		1.002	0.969–1.036	0.921	1.000	0.966–1.034	0.983			
	G1–3 vs. G0					1.986	0.826–4.773	0.125			
	*TLR4* rs7037117	A allele carriers vs. A allele non-carriers	3.762	0.938–15.092	0.062	4.244	1.029–17.501	0.046			
	Age at diagnosis		1.002	0.969–1.036	0.922	1.000	0.966–1.034	0.984			
	G1–3 vs. G0					1.983	0.827–4.757	0.125			
	*TLR4* rs7037117	G allele carriers vs. G allele non-carriers	0.800	0.425–1.506	0.489	0.806	0.426–1.526	0.508			
	Age at diagnosis		1.002	0.969–1.036	0.903	1.000	0.967–1.034	0.984			
	G1–3 vs. G0					1.784	0.763–4.172	0.182			

Abbreviations: OR, odds ratio; CI, confidence interval; x, not applicable.

**Table 5:** Log-rank, Breslow, and Tarone–Ware tests for comparison of relapse-free survival rates by *MYD88* rs7744 genotype distribution.

	Chi-Square	df *	*p*
Log-Rank (Mantel–Cox)	7.892	2	0.019
Breslow (Generalized Wilcoxon)	8.488	2	0.014
Tarone–Ware	8.488	2	0.014

* df—degrees of freedom.

**Table 6:** Log-rank, Breslow, and Tarone–Ware tests for comparison of relapse-free survival rates by *TLR4* rs7037225 genotype distribution.

	Chi-Square	df *	*p*
Log-Rank (Mantel–Cox)	7.275	2	0.026
Breslow (Generalized Wilcoxon)	7.849	2	0.020
Tarone–Ware	8.042	2	0.018

* df—degrees of freedom.

**Table 7:** Cox regression analysis of RFS in patients with LSCC.

Factors		RFS									
		Model I		Model II		Model III		Model IV		Model V	
		HR (95% CI)	*p*	HR (95% CI)	*p*	HR (95% CI)	*p*	HR (95% CI)	*p*	HR (95% CI)	*p*
*TLR4* rs7037225	CT vs. CC	0.119 (0.016–0.870)	0.036	0.120 (0.016–0.881)	0.037	0.120 (0.016–0.877)	0.037	0.100 (0.014–0.736)	0.024	0.108 (0.015–0.795)	0.029
	TT vs. CC	2.165 (0.294–15.937)	0.448	2.339 (0.312–17.508)	0.408	2.320 (0.310–17.366)	0.413	3.856 (0.491–30.294)	0.199	4.022 (0.499–32.419)	0.191
Age at diagnosis				1.012 (0.975–1.051)	0.531	1.012 (0.974–1.052)	0.529	1.008 (0.969–1.049)	0.682	1.009 (0.969–1.051)	0.653
T3–4 vs. T1–2						1.156 (0.585–2.287)	0.676	0.642 (0.284–1.450)	0.286	0.644 (0.273–1.520)	0.315
N1–3 vs. N0								3.197 (1.392–7.343)	0.006	3.109 (1.289–7.496)	0.012
G1–3 vs. G0										0.771 (0.312–1.901)	0.572
*TLR4* rs11536889	GC vs. GG	0.717 (0.277–1.855)	0.492	0.720 (0.278–1.864)	0.498	0.725 (0.280–1.878)	0.508	0.666 (0.257–1.724)	0.402	0.682 (0.262–1.774)	0.433
	CC vs. GG	x	x	x	x	x	x	x	x	x	x
Age at diagnosis				0.534 (0.975–1.050)	0.534	1.012 (0.975–1.050)	0.526	1.008 (0.970–1.047)	0.686	1.010 (0.971–1.050)	0.621
T3–4 vs. T1–2						1.112 (0.562–2.200)	0.760	0.668 (0.294–1.520)	0.336	0.701 (0.297–1.656)	0.418
N1–3 vs. N0								2.587 (1.141–5.867)	0.023	2.437 (1.037–5.728)	0.041
G1–3 vs. G0										0.760 (0.308–1.889)	0.555
*TLR4* rs7037117	AG vs. AA	0.556 (0.251–1.233)	0.149	0.563 (0.253–1.250)	0.158	0.563 (0.254–1.251)	0.159	0.518 (0.232–1.153)	0.107	0.551 (0.246–1.236)	0.148
	GG vs. AA	0.546 (0.074–4.042)	0.554	0.546 (0.074–4.039)	0.553	0.533 (0.072–3.961)	0.539	0.624 (0.083–4.664)	0.646	0.654 (0.087–4.919)	0.680
Age at diagnosis				1.011 (0.974–1.050)	0.560	1.011 (0.974–1.050)	0.553	1.008 (0.969–1.048)	0.701	1.009 (0.970–1.050)	0.655
T3–4 vs. T1–2						1.147 (0.579–2.273)	0.693	0.697 (0.309–1.570)	0.383	0.718 (0.306–1.683)	0.446
N1–3 vs. N0								2.631 (1.168–5.924)	0.020	2.494 (1.063–5.850)	0.036
G1–3 vs. G0										0.792 (0.320–1.961)	0.615
*MYD88* rs7744	AG vs. AA	2.071 (1.029–4.166)	0.041	2.080 (1.033–4.188)	0.040	2.076 (1.031–4.180)	0.041	2.171 (1.073–4.392)	0.031	2.177 (1.067–4.442)	0.033
	GG vs. AA	4.778 (1.100–20.759)	0.037	4.687 (1.078–20.373)	0.039	4.704 (1.081–20.465)	0.039	4.307 (0.986–18.812)	0.052	4.426 (1.003–19.529)	0.050
Age at diagnosis				1.013 (0.974–1.054)	0.513	1.014 (0.974–1.055)	0.499	1.009 (0.969–1.051)	0.664	1.011 (0.970–1.054)	0.598
T3–4 vs. T1–2						1.131 (0.571–2.240)	0.725	0.620 (0.256–1.500)	0.289	0.674 (0.271–1.678)	0.397
N1–3 vs. N0								2.714 (1.127–6.533)	0.026	2.444 (0.987–6.047)	0.053
G1–3 vs. G0										0.785 (0.318–1.935)	0.599
*MYD88* rs6853	AG vs. AA	0.778 (0.351–1.723)	0.539	0.794 (0.357–1.756)	0.572	0.807 (0.361–1.808)	0.603	0.759 (0.336–1.713)	0.506	0.740 (0.323–1.694)	0.476
	GG vs. AA	x	x	x	x	x	x	x	x	x	x
Age at diagnosis				1.001 (0.974–1.050)	0.550	1.012 (0.974–1.051)	0.542	1.008 (0.970–1.048)	0.960	1.010 (0.971–1.051)	0.627
T3–4 vs. T1–2						1.111 (0.559–2.211)	0.764	0.647 (0.274–1.524)	0.319	0.676 (0.274–1.664)	0.394
N1–3 vs. N0								2.605 (1.122–6.048)	0.026	2.464 (1.021–5.947)	0.045
G1–3 vs. G0										0.735 (0.296–1.824)	0.507

Abbreviations: HR, hazard ratio; CI, confidence interval; T, tumor size; N, lymph node with metastasis; G, tumor grade; RFS, relapse-free survival. x—not applicable.

## Data Availability

The data presented in this study are available upon request from the corresponding author due to patient privacy and ethical restrictions.

## Supplementary Materials

The following supporting information can be downloaded at: https://www.mdpi.com/article/10.3390/ijms27114760/s1.

## Abbreviations

The following abbreviations are used in this manuscript:

LSCC	Laryngeal squamous cell carcinoma
SNP	Single-nucleotide polymorphism
TLR	Toll-like receptor
PAMPs	Pathogen-associated molecular patterns
DAMPs	Damage-associated molecular patterns
TIR	Toll/IL-1 receptor
MyD88	Myeloid differentiation primary adapter–response 88
TRIF	TIR domain-containing adapter-inducing interferon- β
LPS	Lipopolysaccharide
PCR	Polymerase chain reaction
LUHS	Lithuanian University of Health Sciences
CT	Computed tomography
MRI	Magnetic resonance imaging
NCCN	National Comprehensive Cancer Network
T	Tumor size
N	Nodal metastasis
M	Distant metastasis
ST	Clinical stage
G	Cancer cell differentiation grade
MAF	Minimal allele frequency
SPSS	Statistical Package for the Social Sciences
HWE	Hardy–Weinberg equilibrium
OR	Odds ratio
CI	Confidence interval
OS	Overall survival
RFS	Relapse-free survival
