# Supplementary material for: Impact of TLR4 and MYD88 Genetic Variants on Disease Progression and Prognosis in Laryngeal Squamous Cell Carcinoma
Source: Int J Mol Sci. 2026 May 25;27(11):4760. doi: 10.3390/ijms27114760 (PMC13256836; doi:10.3390/ijms27114760)
Supplement: Supplementary file 1 [file ijms-27-04760-s001.zip › ijms-4313012-supplementary.pdf]

Supplementary Materials

This file contains supplementary tables and figures of *TLR4* rs11536889, rs7037117 and rs7037225, *MYD88* rs6853 and rs7744 survival analysis which were not presented in the main article. In survival analysis, associations between the tested polymorphisms and overall survival (OS) as well as relapse-free survival (RFS) were evaluated. Survival curves were generated using the Kaplan-Meier method and compared using the log-rank, Breslow, and Tarone-Ware tests. Statistical significance was defined as a value of less than 0.05.

Relapse-free survival (RFS) data

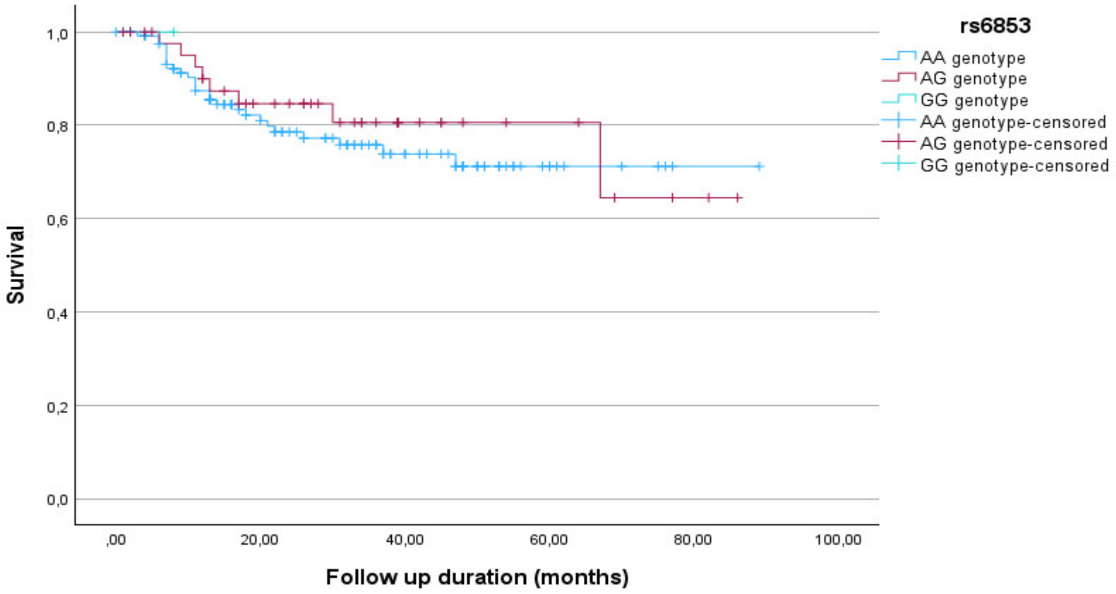

Figure S1. Relapse-free survival rate according to the distribution of *MYD88* rs6853 genotypes.

Table S1. Log-rank, Breslow, and Tarone-Ware tests for relapse-free survival rate comparison according to the distribution of *MYD88* rs6853 genotypes.

	Chi-Square	df*	P-value
Log Rank (Mantel-Cox)	0.458	2	0.796
Breslow (Generalized Wicoxon)	0.636	2	0.728
Tarone-Ware	0.610	2	0.737

*df - degrees of freedom

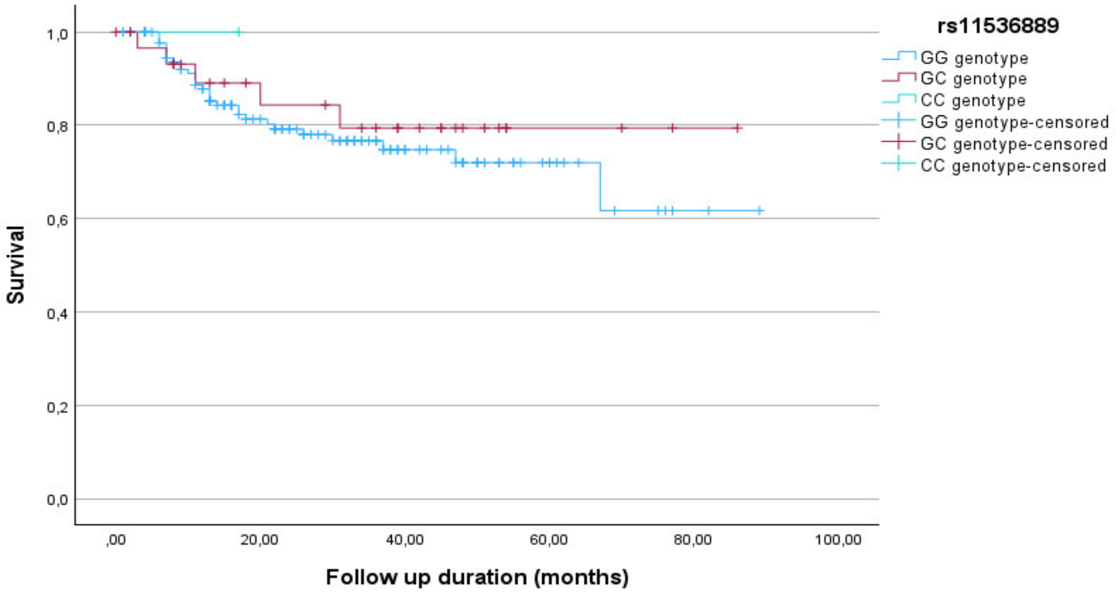

Figure S2. Relapse-free survival rate according to the distribution of *TLR4* rs11536889 genotypes.

Table S2. Log-rank, Breslow, and Tarone-Ware tests for relapse-free survival rate comparison according to the distribution of *TLR4 rs11536889* genotypes.

	Chi-Square	df*	P-value
Log Rank (Mantel-Cox)	0.663	2	0.718
Breslow (Generalized Wicoxon)	0.411	2	0.814
Tarone-Ware	0.493	2	0.782

*df - degrees of freedom

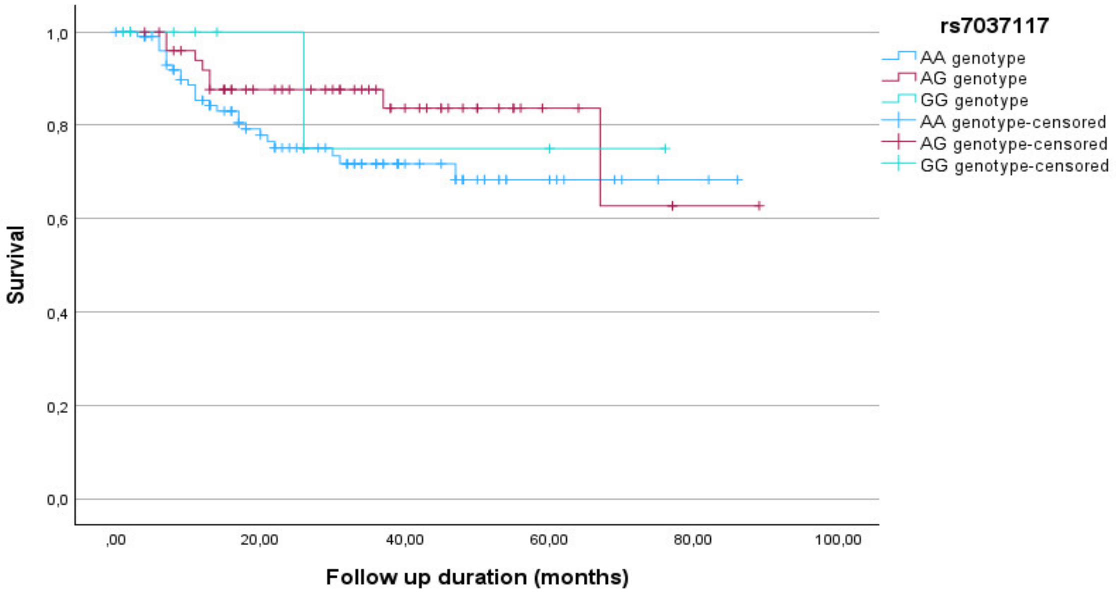

Figure S3. Relapse-free survival rate according to the distribution of *TLR4* rs7037117 genotypes.

Table S3. Log-rank, Breslow, and Tarone-Ware tests for relapse-free survival rate comparison according to the distribution of *TLR4* rs7037117 genotypes.

	Chi-Square	df*	P-value
Log Rank (Mantel-Cox)	2.382	2	0.304
Breslow (Generalized Wicoxon)	2.930	2	0.231
Tarone-Ware	2.856	2	0.240

*df – degrees of freedom

Overall survival (OS) data

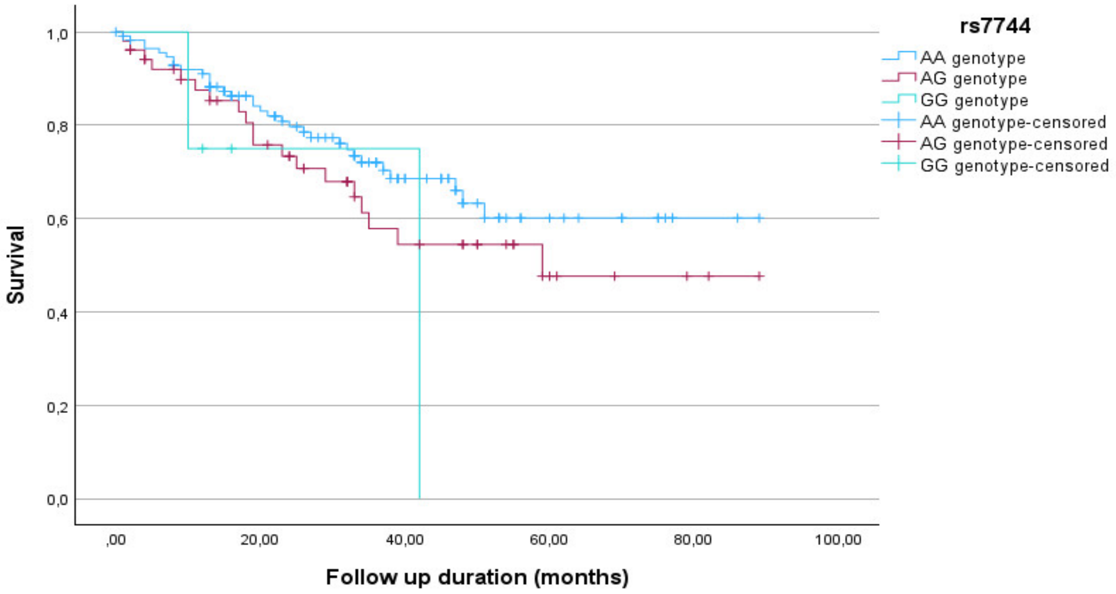

Figure S4. Overall survival rate according to the distribution of *MYD88* rs7744 genotypes.

Table S4. Log-rank, Breslow, and Tarone-Ware tests for overall survival rate comparison according to the distribution of *MYD88* rs7744 genotypes.

	Chi-Square	df*	P-value
Log Rank (Mantel-Cox)	3.097	2	0.213
Breslow (Generalized Wicoxon)	1.852	2	0.396
Tarone-Ware	2.327	2	0.312

*df – degrees of freedom

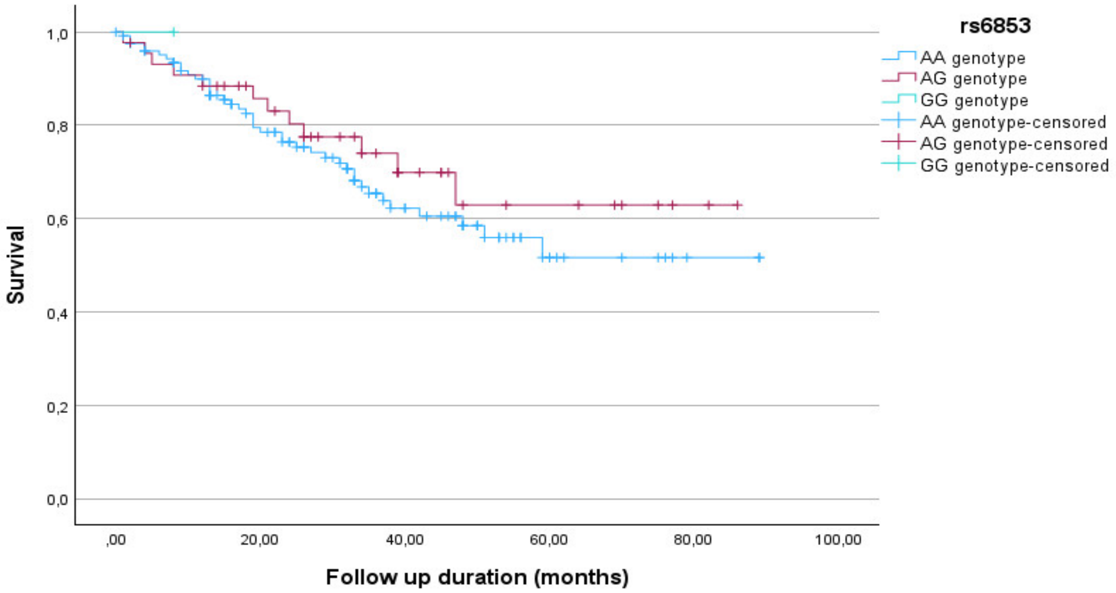

Figure S5. Overall survival rate according to the distribution of *MYD88* rs6853 genotypes.

Table S5. Log-rank, Breslow, and Tarone-Ware tests for overall survival rate comparison according to the distribution of *MYD88* rs6853 genotypes.

	Chi-Square	df*	P-value
Log Rank (Mantel-Cox)	0.793	2	0.673
Breslow (Generalized Wicoxon)	0.543	2	0.762
Tarone-Ware	0.661	2	0.718

*df – degrees of freedom

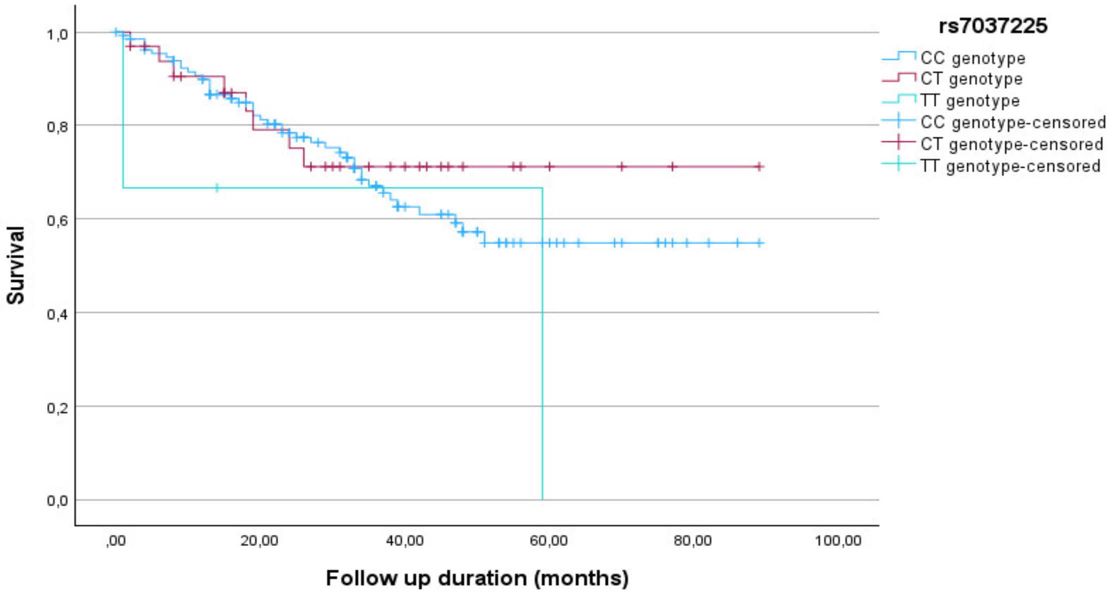

Figure S6. Overall survival rate according to the distribution of *TLR4* rs7037225 genotypes.

Table S6. Log-rank, Breslow, and Tarone-Ware tests for overall survival rate comparison according to the distribution of *TLR4* rs7037225 genotypes.

	Chi-Square	df*	P-value
Log Rank (Mantel-Cox)	2.614	2	0.271
Breslow (Generalized Wicoxon)	1.441	2	0.487
Tarone-Ware	1.575	2	0.455

*df – degrees of freedom

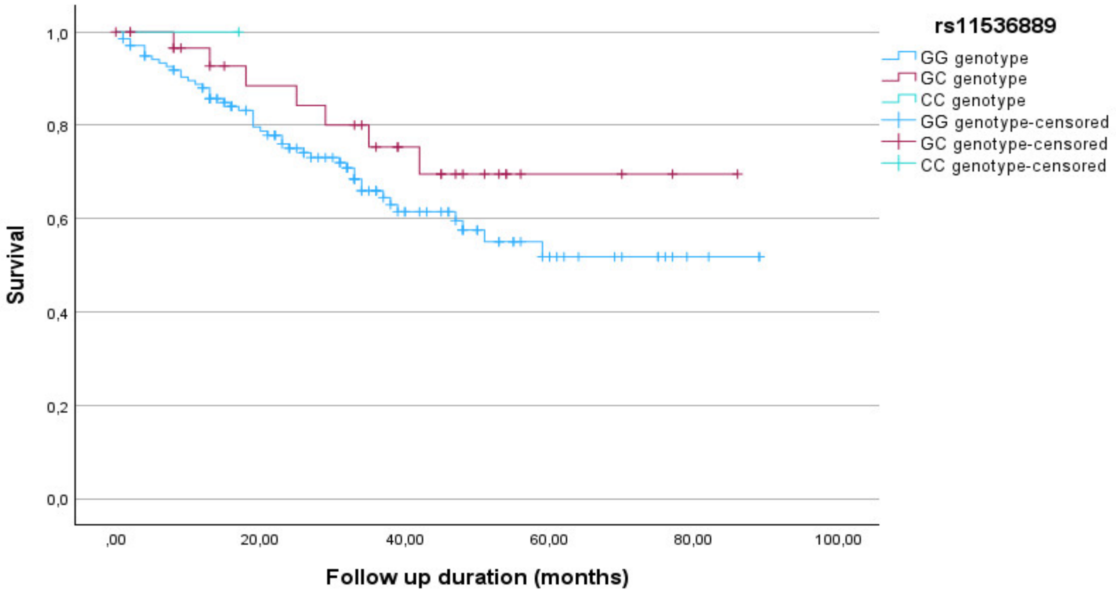

Figure S7. Overall survival rate according to the distribution of *TLR4* rs11536889 genotypes.

Table S7. Log-rank, Breslow, and Tarone-Ware tests for overall survival rate comparison according to the distribution of *TLR4 rs11536889* genotypes.

	Chi-Square	df*	P-value
Log Rank (Mantel-Cox)	1.824	2	0.402
Breslow (Generalized Wicoxon)	1.913	2	0.384
Tarone-Ware	1.879	2	0.391

*df – degrees of freedom

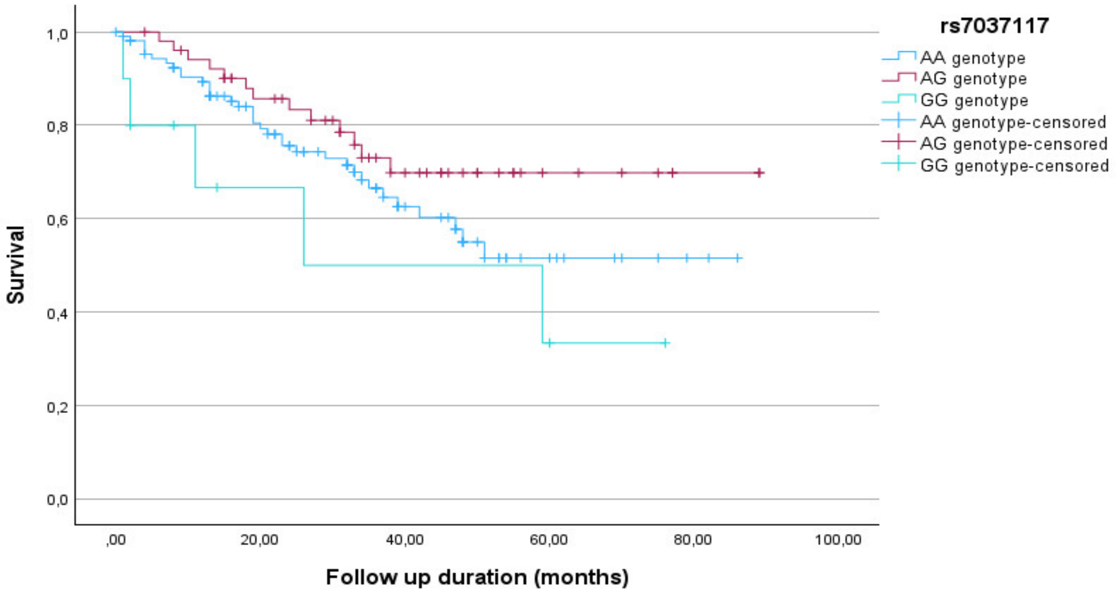

Figure S8. Overall survival rate according to the distribution of *TLR4* rs7037117 genotypes.

Table S8. Log-rank, Breslow, and Tarone-Ware tests for overall survival rate comparison according to the distribution of *TLR4* rs7037117 genotypes.

	Chi-Square	df*	P-value
Log Rank (Mantel-Cox)	4.771	2	0.092
Breslow (Generalized Wicoxon)	5.273	2	0.072
Tarone-Ware	4.890	2	0.087

df – degrees of freedom
